# Surgical Versus Conservative Management of Supratentorial ICH: A Single-Center Retrospective Analysis (2017–2023)

**DOI:** 10.3390/jcm14155372

**Published:** 2025-07-30

**Authors:** Cosmin Cindea, Samuel Bogdan Todor, Vicentiu Saceleanu, Tamas Kerekes, Victor Tudor, Corina Roman-Filip, Romeo Gabriel Mihaila

**Affiliations:** 1 Faculty of Medicine, Lucian Blaga University of Sibiu, 550024 Sibiu, Romaniavicentiu.saceleanu@ulbsibiu.ro (V.S.); corina.roman@ulbsibiu.ro (C.R.-F.); romeo.mihaila@ulbsibiu.ro (R.G.M.); 2 County Clinical Emergency Hospital of Sibiu, 550245 Sibiu, Romania; 3 County Clinical Emergency Hospital of Brasov, 500097 Brasov, Romania

**Keywords:** supratentorial intracerebral hemorrhage, surgical intervention, conservative management, Glasgow Coma Scale, hematoma volume

## Abstract

**Background**: Intracerebral hemorrhage (ICH) is a severe form of stroke associated with high morbidity and mortality. While neurosurgical evacuation may offer theoretical benefits, its impact on survival and hospital course remains debated. We aimed to compare the outcomes of surgical versus conservative management in patients with lobar, capsulo-lenticular, and thalamic ICH and to identify factors influencing mortality and the surgical decision. **Methods**: This single-center, retrospective cohort study included adult patients admitted to the County Clinical Emergency Hospital of Sibiu (2017–2023) with spontaneous supratentorial ICH confirmed via CT (deepest affected structure determining lobar, capsulo-lenticular, or thalamic location). We collected data on demographics, clinical presentation (Glasgow Coma Scale [GCS], anticoagulant use), hematoma characteristics (volume, extension), treatment modality (surgical vs. conservative), and in-hospital outcomes (mortality, length of stay). Statistical analyses included *t*-tests, χ^2^, correlation tests, and logistic regression to identify independent predictors of mortality and surgery. **Results**: A total of 445 patients were analyzed: 144 lobar, 150 capsulo-lenticular, and 151 thalamic. Surgical intervention was more common in patients with larger volumes and lower GCS. Overall, in-hospital mortality varied by location, reaching 13% in the lobar group, 20.7% in the capsulo-lenticular group, and 35.1% in the thalamic group. Within each location, surgical intervention did not significantly reduce overall in-hospital mortality despite the more severe baseline presentation in surgical patients. In lobar ICH specifically, no clear survival advantage emerged, although surgery may still benefit those most severely compromised. For capsulo-lenticular hematomas > 30 mL, surgery was associated with lower mortality (39.4% vs. 61.5%). In patients with large lobar ICH, surgical intervention was associated with mortality rates similar to those seen in less severe, conservatively managed cohorts. Multivariable adjustment confirmed GCS and hematoma volume as independent mortality predictors; age and volume predicted the likelihood of surgical intervention. **Conclusions**: Despite targeting more severe cases, neurosurgical evacuation did not uniformly lower in-hospital mortality. In lobar ICH, surgical patients with larger hematomas (~48 mL) and lower GCS (~11.6) had mortality rates (~13%) comparable to less severe, conservative cohorts, indicating that surgical intervention was associated with similar mortality rates despite higher baseline risk. However, these findings do not establish a causal survival benefit and should be interpreted in the context of non-randomized patient selection. For capsulo-lenticular hematomas > 30 mL, surgery was associated with lower observed mortality (39.4% vs. 61.5%). Thalamic ICH remained most lethal, highlighting the difficulty of deep-brain bleeds and frequent ventricular extension. Across locations, hematoma volume and GCS were the primary outcome predictors, indicating the need for timely intervention, better patient selection, and possibly minimally invasive approaches. Future prospective multicenter research is necessary to refine surgical indications and validate these findings. To our knowledge, this investigation represents the largest and most contemporary single-center cohort study of supratentorial intracerebral hemorrhage conducted in Romania.

## 1. Introduction

Intracerebral hemorrhage (ICH) is a devastating form of stroke, accounting for approximately 10–15% of all stroke cases worldwide, yet contributing disproportionately to stroke-related mortality and morbidity [1].

Despite advances in critical care, ICH remains associated with a one-month mortality rate as high as 40% [2]. In terms of location, lobar ICH—involving bleeding in the cortical–subcortical regions of the cerebral lobes—comprises roughly one-third of spontaneous ICH cases [3]. This lobar subtype in older adults is often linked to cerebral amyloid angiopathy (CAA), whereas deep ICH (e.g., capsulo-lenticular/basal ganglia and thalamic hemorrhages) is more frequently associated with chronic hypertension [4,5]. Capsulo-lenticular hemorrhages (involving the basal ganglia) and thalamic hemorrhages both carry a high risk of neurological deterioration, given their proximity to vital subcortical structures [6]. Meanwhile, CAA-associated lobar ICH not only poses immediate threats (due to mass effect and elevated intracranial pressure), but also carries a high rebleeding risk over time [4,7].

Regardless of location, management of ICH typically begins with aggressive medical therapy, including control of elevated blood pressure, reversal of anticoagulation (when applicable), and comprehensive neurologic support in an intensive care setting [2,8]. However, the potential role of surgical evacuation remains a subject of debate. The theoretical benefits of surgery include removal of toxic blood breakdown products, relief of intracranial pressure, and possible mitigation of secondary brain injury [9]. However, large randomized trials—notably the STICH (Surgical Trial in Intracerebral Hemorrhage) series—have yielded equivocal results, showing no clear survival or functional advantage for early hematoma evacuation over best medical care [9,10]. Meta-analyses investigating both open craniotomy and minimally invasive surgical (MIS) approaches have likewise been inconclusive, though certain subgroups (e.g., patients with relatively superficial lobar clots) or large capsulo-lenticular hemorrhages might demonstrate improved outcomes [1,11]. Thalamic ICH, by contrast, has proven particularly challenging given its deep location and high risk of ventricular extension, further complicating surgical decisions [6,12]. However, most large-scale studies have focused on broad patient populations, and there remains a need for single-center, real-world analyses that can provide more granular insights into local surgical selection patterns and outcomes, especially in resource-constrained environments.

Recent efforts have focused on refining patient selection and exploring innovative surgical methods, especially in deep-seated hematomas. Minimally invasive techniques, such as endoscopic clot evacuation, stereotactic aspiration, or catheter-based thrombolysis, aim to reduce procedure-related trauma while decompressing the hematoma [11,13]. For instance, the MISTIE III trial demonstrated that MIS can be performed safely for moderate-to-large ICH, but did not establish a significant improvement in long-term functional outcomes compared with conservative management [13]. Other ongoing trials, such as ENRICH, continue to investigate novel surgical approaches that could alter the current standard of care [14]. Although guidelines offer cautious endorsements for surgery—recommending it primarily for large, accessible hemorrhages with clinical deterioration or mass effect—many centers vary in their thresholds, especially when factoring in location (lobar vs. basal ganglia vs. thalamus), anticoagulation status, and patient comorbidities [2,8]. Though large-scale randomized trials such as STICH and MISTIE have examined the role of surgery in intracerebral hemorrhage, it remains uncertain how single-center data can elucidate local practice patterns and real-world treatment decisions.

Recent clinical guidelines, including the 2022 American Heart Association (AHA) and the 2025 European Stroke Organisation (ESO) recommendations, highlight the complexity of surgical decision-making in supratentorial ICH. Both advise against routine hematoma evacuation and favor individualized patient selection based on clinical and radiological criteria. However, they do not specify which anatomical locations—such as lobar, capsulo-lenticular, or thalamic—are likely to benefit from surgery. This lack of location-specific guidance underscores the need for stratified observational data to inform real-world practices and refine surgical indications [15,16].

Given these complexities, real-world observational data are essential to clarifying how clinicians choose surgical vs. conservative management for each ICH location and how such decisions impact patient outcomes. In this single-center retrospective study, we examined short-term clinical outcomes—specifically in-hospital mortality and length of stay—among lobar, capsulo-lenticular, and thalamic ICH patients who were either surgically or conservatively managed at a tertiary referral hospital over a seven-year period. We also assessed the influence of age, hematoma volume, neurological status, and anticoagulant use on treatment decisions and prognoses. By comparing these three ICH locations within a single institutional experience, our study aims to refine current treatment discussions, identify potential subgroups that may benefit most from surgical intervention, and highlight persistent controversies that limit consensus on optimal therapy for spontaneous ICH.

While randomized controlled trials have provided important insights into supratentorial ICH management, there is a paucity of large-scale, contemporary observational data from Central and Eastern Europe. The present study addresses this gap by analyzing the most extensive and up-to-date cohort of patients with supratentorial ICH reported in Romania. Conducted at a major tertiary referral center, our study reflects institutional protocols that align with internationally recognized standards, including 24/7 computed tomography imaging availability and immediate access to open surgical evacuation.

Although large multicenter trials like STICH [9,10] and MISTIE [13] have advanced our understanding of surgical management of ICH, they raise unresolved questions about location-specific outcomes. Lobar hemorrhages, commonly linked to cerebral amyloid angiopathy, differ pathophysiologically and in procedural feasibility from deep-seated capsulo-lenticular and thalamic bleeds, which stem primarily from hypertensive vasculopathy. By stratifying patients into distinct anatomical groups, our single-center study offers more focused insights into real-world surgical decision making and outcomes. This nuanced approach may clarify which subgroups derive the greatest benefit from operative intervention, thereby refining the broader lessons from STICH and MISTIE and guiding more precise, location-aware treatment strategies.

We hypothesized that, in a real-world, single-center setting, surgery might provide outcome benefits for certain ICH subtypes, given careful patient selection based on hematoma volume and GCS.

## 2. Materials and Methods

### 2.1. Study Design and Ethical Considerations

This single-center, retrospective cohort study was conducted at the County Clinical Emergency Hospital of Sibiu, a tertiary referral center serving both urban and rural populations. All adult patients (≥18 years) diagnosed with spontaneous intracerebral hemorrhage (ICH) between January 2017 and December 2023 were screened. Only supratentorial (lobar, capsulo-lenticular (basal ganglia), or thalamic) ICH were included (classified by the deepest affected anatomical structure). As treatment allocation was determined by the attending neurosurgeon based on clinical criteria (hematoma volume, GCS, comorbidities), selection bias is possible. The Sibiu Emergency Hospital Ethics Committee approved the study (Approval Code: SEH-R-87/2023), and the need for individual patient consent was waived due to the retrospective design of the study.

### 2.2. Inclusion and Exclusion Criteria

Inclusion: (1) spontaneous ICH confirmed via non-contrast CT; (2) admission within 48 h of symptom onset or sufficient documentation of presentation; (3) age ≥ 18.

Exclusion: (1) traumatic ICH or secondary hemorrhages (e.g., arteriovenous malformation); (2) primary infratentorial hemorrhages; (3) incomplete records preventing reliable assessment (e.g., unknown admission GCS or hemorrhage volume).

### 2.3. Data Collection

Demographic and Clinical: age, sex, rural/urban residence, admission GCS, anticoagulant use, comorbidities, time from onset to admission.

Imaging: ICH location (lobar, capsulo-lenticular, thalamic), hemorrhage volume (ABC/2 method), intraventricular extension.

Treatment: surgical (open craniotomy or minimally invasive) vs. conservative.

Treatment decisions followed institutional protocols guided by recent ESO [16] and AHA [15] guidelines, emphasizing individualized patient selection based on clinical presentation and hematoma characteristics.

Outcomes: in-hospital mortality, length of stay (LOS), discharge destination, and major complications.

### 2.4. Management Protocols

All patients received standard ICH care as per international guidelines [12,13], including blood pressure control (target systolic < 140 mmHg), coagulopathy reversal if anticoagulated, and ICU monitoring for large hemorrhages or severe neurologic compromise (e.g., GCS < 8). Surgical evacuation was considered for large (>30–40 mL), accessible hematomas causing clinical deterioration or mass effect, with final decisions at the attending neurosurgeon’s discretion. These criteria were generally consistent with the principles outlined in the AHA 2022 and ESO guidelines, which recommend individualized treatment based on hematoma size, neurologic status, and imaging features.

### 2.5. Statistical Analysis

Data were analyzed using SPSS v.26 (IBM, Armonk, NY, USA). Continuous variables were assessed with the Student’s *t*-test or Mann–Whitney U-test, and categorical variables with the chi-square or Fisher’s exact test, as appropriate. Multivariable logistic regression was used to identify independent predictors of in-hospital mortality and surgical intervention, including age, admission GCS, hematoma volume, anticoagulant use, and ICH location. An interaction analysis between hematoma location and volume > 30 mL was conducted to evaluate whether surgical outcomes differed by anatomical site in large hematomas. Subgroup mortality rates were adjusted for key covariates to assess real-world prognostic trends. A *p*-value < 0.05 was considered statistically significant.

### 2.6. Data Availability and Use of Generative AI

De-identified data are available from the corresponding author upon reasonable request, subject to ethical approval. No generative AI tools were used to analyze data or produce scientific content; AI-based language models contributed only minor grammatical edits.

## 3. Results

**Figure 1:**
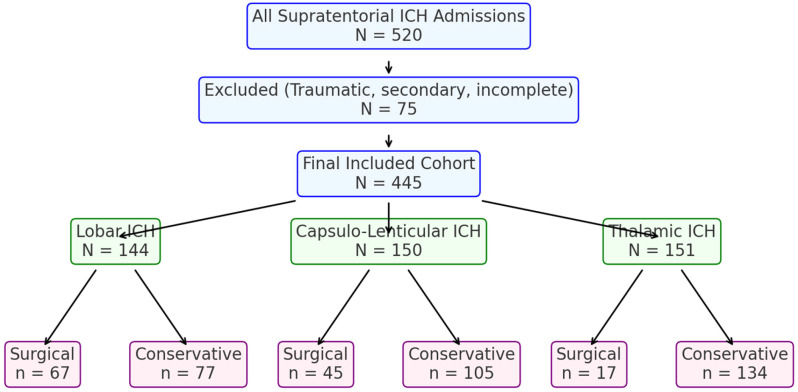
Flow diagram of patient inclusion and treatment allocation.

A total of 445 adult patients with supratentorial intracerebral hemorrhage (ICH) were included in this retrospective analysis at the County Clinical Emergency Hospital of Sibiu from 2017 to 2023. They were categorized into three groups by hemorrhage location: lobar (*n* = 144), capsulo-lenticular (*n* = 150), and thalamic (*n* = 151). Overall management involved surgical evacuation (via open craniotomy) or conservative (medical) treatment. Primary considerations for management included hematoma volume, admission Glasgow Coma Scale (GCS), and anticoagulant/antiplatelet status (Figure 1).

In the lobar ICH subgroup (*n* = 144), 67 (46.5%) underwent surgical evacuation, and 77 (53.5%) were managed conservatively. Table 1 displays their demographic and clinical data. Surgical patients had a younger mean age (67.6 vs. 72.2 years, *p* < 0.001), larger mean hematoma volume (48.0 vs. 19.4 mL, *p* < 0.001), and lower admission GCS (11.6 vs. 13.1, *p* < 0.001). Anticoagulant or antiplatelet use was more frequent in the conservative group (24.7% vs. 10.4%, *p* = 0.046). In-hospital mortality was 13.4% among surgical cases and 13.0% among conservative cases. The 30-day mortality was 31.25% for the surgical group and 24.7% for the conservative group (*p* = 0.32). The mean length of stay (LOS) was longer for surgically treated patients (15 vs. 11 days), with *p*-values ranging from <0.001 to 0.03 in different subgroup analyses.

Capsulo-Lenticular ICH (*n* = 150). Of these, 45 patients (30.0%) underwent surgical evacuation, whereas 105 (70.0%) received conservative treatment. Surgically managed patients had a larger mean hematoma volume (68.9 vs. 18.1 mL, *p <* 0.001) and a lower admission GCS (9.35 vs. 12.48). Unadjusted mortality was 33.3% in the surgical group and 15.2% in the conservative group (*p* = 0.01). In a subset of 46 patients with hematoma volumes > 30 mL, mortality was 39.4% in the surgical cohort (n = 33) compared with 61.5% in the conservative cohort (n = 13). Anticoagulation was more common in conservatively treated individuals (23 vs. 5), and the mean length of stay (LOS) was 13 days (surgical) versus 14 days (conservative) (*p* = 0.65).

Thalamic ICH (*n* = 151). Seventeen patients (11%) received surgical intervention, while 134 (89%) underwent conservative management. The surgical subgroup had a larger mean hematoma volume (63.8 vs. 20.7 mL) and a lower admission GCS (6.12 vs. 10.8). In-hospital mortality was 82.3% for the surgically treated group versus 29.9% for the conservative group (*p <* 0.001). The mean LOS was 11 days (surgical) and 13 days (conservative). Anticoagulant use was observed in 3 surgical patients compared with 35 in the conservative cohort.

Across all ICH locations, patients selected for surgery typically had larger hematoma volumes (≥30 mL in lobar ICH and often >60 mL in capsulo-lenticular and thalamic ICH), with volumes approaching ~70 mL in severe capsulo-lenticular cases. Admission GCS was also lower in surgical cohorts. In the thalamic subgroup, for example, a mean GCS of 6.12 corresponded to an 82.3% mortality among surgically treated patients.

Anticoagulation status varied by treatment approach. In the lobar subgroup, 24.7% of conservatively managed patients were anticoagulated compared with 10.4% in the surgical group (*p* = 0.046). Similarly, in capsulo-lenticular ICH, 23 conservative patients versus 5 surgical patients were on anticoagulants, whereas in thalamic ICH, 35 conservative patients compared with 3 surgical patients had anticoagulation.

Age, comorbidities, and geographic origin also influenced outcomes. Older individuals and those with diabetes mellitus were more often managed conservatively. In lobar ICH, 13% of conservative patients had diabetes versus 4.5% of surgical patients. Overall, rural residence was noted in 57 of 144 lobar, 65 of 150 capsulo-lenticular, and 76 of 151 thalamic cases. Among 31 total capsulo-lenticular deaths, 9 were rural residents; among 53 thalamic deaths, 22 were rural. However, no direct correlation between rural origin and mortality was established.

**Figure 2:**
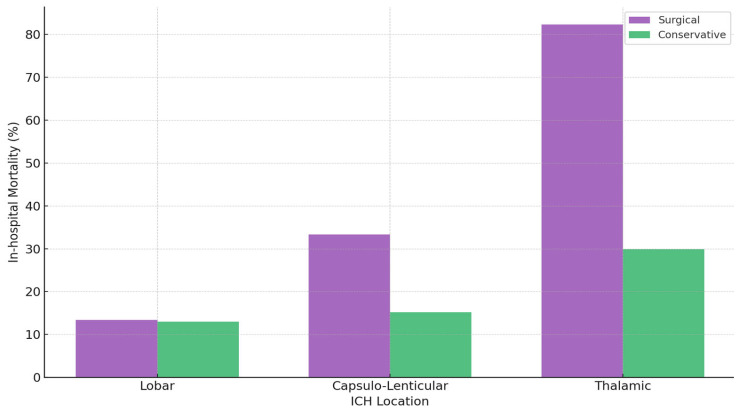
Mortality rates by ICH location and treatment type.

A comparative overview of surgical versus conservative outcomes by location (Table 2) shows that lobar ICH has relatively favorable survival (~13% mortality), with no clear surgical advantage when data are unadjusted for severity. Capsulo-lenticular ICH exhibits moderate overall mortality (~20.7%), rising to 33.3% in the surgical group unless large-volume cases (>30 mL) are isolated, where lower observed mortality was seen in the surgical group (39.4% vs. 61.5%). Thalamic ICH has the poorest outcomes in surgically treated patients (82.3% mortality), reflecting the severity of high-volume bleeds with profoundly low GCS. Length of hospital stay also varies by location: surgical lobar cases stay longer (15 vs. 11 days), capsulo-lenticular groups differ minimally (13 vs. 14 days), and surgical thalamic patients often have shorter stays due to earlier mortality (11 vs. 13 days) (Figure 2).

Multivariable logistic regression identified admission GCS and hematoma volume as the strongest independent predictors of in-hospital mortality (*p <* 0.001). Surgical treatment was associated with lower mortality in capsulo-lenticular ICH > 30 mL (39.4% vs. 61.5%, *p* = 0.04), but not in thalamic ICH, where mortality remained high despite intervention (82.3% vs. 29.9%, *p <* 0.001). In lobar ICH, mortality was similar between surgical and conservative groups (13.4% vs. 13.0%, *p* = 0.94); yet surgical patients had significantly larger hematomas (48.0 vs. 19.4 mL, *p <* 0.001) and lower GCS (11.6 vs. 13.1, *p <* 0.001), suggesting a potential risk-adjusted benefit of surgery—consistent with selective recommendations from the AHA 2022 and ESO 2025 guidelines. Overall, hematoma location influenced outcomes, particularly in large-volume deep ICH, while factors such as anticoagulant use, age, and comorbidities added complexity without excluding surgical consideration.

## 4. Discussion

In this single-center retrospective study, we evaluated short-term outcomes—namely in-hospital mortality and length of stay—among patients with lobar, capsulo-lenticular, and thalamic intracerebral hemorrhage (ICH). Surgical management generally targeted those with larger hematomas and lower Glasgow Coma Scale (GCS) scores, aiming to mitigate the higher risk of severe presentations. Even so, mortality rates in certain surgical groups were not markedly different from those of conservative groups, indicating that, in this cohort, surgical intervention was associated with similar outcomes despite greater baseline severity.

In the lobar ICH cohort, the surgically treated patients had significantly larger hematomas (~48 mL) and lower GCS (~11.6), but showed in-hospital mortality (~13%) comparable to less severe, conservative cases. These findings align with prior large trials indicating no definitive advantage in terms of mortality for open craniotomy in unselected supratentorial hemorrhages; however, in our cohort, surgery was associated with similar in-hospital mortality despite greater hematoma size [17,18]. Moreover, advanced age and anticoagulant use often shifted management toward conservative therapy, reflecting evidence that coagulopathy and frailty heighten perioperative risk [19]. As our study was retrospective and non-randomized, selection bias (whereby sicker patients were chosen for surgery) may limit direct outcome comparisons.

For capsulo-lenticular ICH, we observed higher overall mortality (~33%) in the surgical group than in the conservative group (~15%), likely reflecting more severe baseline status in operated patients. However, among large capsulo-lenticular clots (>30 mL), mortality was lower with surgery (39.4%) than with conservative treatment (61.5%). This aligns with reports suggesting that selected deep-seated ICH may benefit from targeted surgical removal, particularly when minimally invasive methods are employed [20,21,22,23].

Our findings align with the 2025 ESO and 2022 AHA guidelines, which discourage routine surgery for supratentorial ICH and recommend individualized decisions based on hematoma volume, neurological status, and feasibility. Both guidelines cautiously support surgery for selected lobar ICH, though without definitive evidence. Our data suggest a potential benefit of surgical evacuation in lobar hemorrhages with large volume and low GCS, consistent with these selective recommendations. Additionally, we observed improved survival in capsulo-lenticular ICH > 30 mL, while thalamic ICH remained associated with high mortality regardless of treatment. These results offer location-specific insights that may help refine surgical indications beyond the current guideline scope [15,16].

Thalamic ICH emerged as the most lethal subtype, especially among surgical candidates. This finding is consistent with its deep location, high risk of ventricular extension, and severe neurological compromise (mean GCS ~6.1) [24,25]. Although some guidelines cautiously endorse surgery for large, deteriorating thalamic bleeds, many centers remain hesitant due to technical challenges and the potential for abrupt neurologic decline [26]. Subgroup analyses in the thalamic group (n = 17 surgical patients) were limited by small numbers, and these findings should be interpreted with caution.

Across all three sites, older age and anticoagulant use correlated with conservative management, consistent with the heightened bleeding hazards in these populations [19]. Several surgical fatalities involved patients from rural areas, hinting at possible delays in presentation or limited resources. Future prospective registries could clarify how socioeconomic and geographic factors influence ICH severity and outcomes. Nevertheless, this real-world, single-center cohort offers pragmatic treatment decisions outside controlled trial settings, providing valuable data on everyday clinical practice.

Additional considerations include the timing of intervention. While early evacuation (within 24–48 h) may reduce hematoma expansion, the ideal window is debated, as delayed surgery can lead to worsened edema and clot organization [27]. Advanced imaging protocols, such as MRI or perfusion CT, may help to identify salvageable tissue or predict hematoma growth, enabling more precise treatment decisions [28]. Cost-effectiveness analyses are also needed, given the substantial resources required for critical care, surgery, and rehabilitation in large hemorrhages, particularly in lower-resource settings [29]. Finally, long-term functional outcomes deserve further study. Although immediate survival is crucial, survivors often face extensive rehabilitation, risk of rebleeding, and reduced quality of life, underscoring the need for standardized assessments such as the modified Rankin Scale [30].

Overall, our findings suggest that surgery for large, clinically severe ICH may help balance a high baseline risk, even if it does not uniformly lower mortality. Refining surgical indications and weighing factors such as timing, imaging, and comorbidities could improve risk stratification and tailor interventions to those most likely to benefit over conservative management.

### Limitations

This single-center, retrospective study offers real-world data on ICH management but is subject to several methodological limitations. The absence of standardized long-term functional assessments, such as the modified Rankin Scale, constrains evaluation of post-discharge recovery and neurological outcomes. The non-randomized nature of treatment allocation introduces the potential for selection bias and unmeasured confounding variables. Additionally, heterogeneity in clinical presentation and timing of intervention further limits the ability to draw causal inferences from observed associations. Prospective, multicenter studies employing rigorous patient selection criteria and standardized outcome measures are warranted to validate and expand upon these findings.

The external validity of our results is influenced by several contextual factors unique to both our institution and the Romanian patient population. As a middle–high-income country, Romania is characterized by distinct demographic and clinical features, including specific comorbidity profiles, risk factors, and a life expectancy marginally lower than that observed in high-income nations. These regional characteristics may shape both clinical presentation and management outcomes and should be considered when interpreting our findings internationally. Furthermore, as a tertiary referral center, our hospital receives a disproportionate number of patients with larger or more clinically severe hematomas, often due to delayed presentation. Our management protocols are aligned with internationally recognized standards for acute ICH care, including continuous CT imaging, a dedicated neurosurgery department, and routine access to open surgical evacuation as the global standard. Nevertheless, the limited implementation of minimally invasive surgical techniques in our setting may affect the comparability of outcomes with centers where such approaches are more prevalent.

## 5. Conclusions

In this single-center retrospective study, surgical intervention for lobar, capsulo-lenticular, and thalamic ICH primarily targeted patients with larger hematomas and lower GCS scores. While causality cannot be inferred due to non-randomized selection, our findings suggest a potential **risk-adjusted benefit of surgery in lobar ICH**, where patients with larger clots (~48 mL) and lower GCS (~11.6) had similar mortality (~13%) to conservatively managed cohorts. In **capsulo-lenticular ICH > 30 mL**, surgery was associated with reduced mortality (39.4% vs. 61.5%), indicating **benefit in select high-risk cases**. **Thalamic ICH showed poor outcomes** regardless of treatment, reflecting its severity and anatomical complexity. Overall, hematoma volume and GCS remained key outcome predictors, underscoring the need for timely, individualized management and prospective validation.

These findings underscore the need for prospective multicenter trials to refine surgical indications, validate location-specific strategies, and assess the role of minimally invasive techniques and advanced imaging in optimizing care for spontaneous ICH.

## Figures and Tables

**Table 1:** Demographic and clinical characteristics of patients with lobar ICH (*n* = 144).

Variable	Surgical (*n* = 67)	Conservative (n = 77)	*p*-Value
Age (years), mean ± SD	67.6	72.2	<0.001
Gender, n (%)			
Female	29 (43%)	41 (53%)	0.28
Male	38 (57%)	36 (47%)	
Rural Provenance, n (%)	26 (39%)	31 (40%)	0.51
Admission GCS, mean ± SD	11.6	13.1	<0.001
Anticoagulant/Antiplatelet use, n (%)	7 (10.4%)	19 (24.7%)	0.046
Diabetes Mellitus, n (%)	3 (4.5%)	10 (13.0%)	0.06
Rebleeding, n (%)	~5%	~7%	0.55
30-Day Mortality, n (%)	~31.25%	24.7%	0.32
Type of Surgery	100% Open	Not applicable	—
Surgical Site Infection, n (%)	0%	Not applicable	—

**Table 2:** Comparative analysis of surgical vs. conservative management by ICH location.

ICH Location	Group	Patients (n)	Mean Volume (mL)	Mortality (%)	Mean LOS (days)	Statistical Significance (Surgical vs. Conservative)
Lobar (*n* = 144)	Surgical	67	48.0	13.4	15	Volume: ***p <* 0.001** Mortality: *p* = 0.94 LOS: ***p* = 0.03**
	Conservative	77	19.4	13.0	11	
Capsulo-Lenticular (*n* = 150)	Surgical	45	68.9	33.3	13	Volume: ***p <* 0.001** Mortality: ***p* = 0.01** LOS: *p* = 0.65
	Conservative	105	18.1	15.2	14	
Thalamic (*n* = 151)	Surgical	17	63.8	82.3	11	Volume: ***p <* 0.001** Mortality: ***p <* 0.001** LOS: *p* = 0.21
	Conservative	134	20.7	29.9	13	

LOS = Length of Hospital Stay; significant values (*p <* 0.05) highlighted in bold.

## Data Availability

The patient database supporting the findings of this study is publicly available on Zenodo and can be accessed at the following link: https://doi.org/10.5281/zenodo.15299709 [31].

## Abbreviations

The following abbreviations are used in this manuscript:

ICH	Intracerebral Hemorrhage
GCS	Glasgow Coma Scale
CT	Computed Tomography
MRI	Magnetic Resonance Imaging
LOS	Length of Stay
MIS	Minimally Invasive Surgery
ICU	Intensive Care Unit
CAA	Cerebral Amyloid Angiopathy
STICH	Surgical Trial in Intracerebral Hemorrhage
AHA/ASA	American Heart Association/American Stroke Association
